# Effect of dynamic random leaks on the monitoring accuracy of home mechanical ventilators: a bench study

**DOI:** 10.1186/1471-2466-13-75

**Published:** 2013-12-10

**Authors:** Ana Sogo, Jaume Montanyà, Eduard Monsó, Lluís Blanch, Xavier Pomares, Manel Lujàn

**Affiliations:** 1 Department of Pneumology, Corporació Sanitària Parc Taulí, Institut Universitari Parc Taulí, Universitat Autònoma de Barcelona, Sabadell, Spain; 2 Bettercare S.L., Sabadell, Spain; 3 CIBERES, Ciber de Enfermedades Respiratorias. Carretera Soller Km 12, Bunyola, Spain; 4 Department of Critical Care, Corporació Sanitària Parc Taulí, Institut Universitari Parc Taulí, Universitat Autònoma de Barcelona, Sabadell, Spain

**Keywords:** Non-invasive ventilation, Tidal volume, Unintentional leaks, Bench

## Abstract

**Background:**

So far, the accuracy of tidal volume (VT) and leak measures provided by the built-in software of commercial home ventilators has only been tested using bench linear models with fixed calibrated and continuous leaks. The objective was to assess the reliability of the estimation of tidal volume (VT) and unintentional leaks in a single tubing bench model which introduces random dynamic leaks during inspiratory or expiratory phases.

**Methods:**

The built-in software of four commercial home ventilators and a fifth ventilator-independent *ad hoc* designed external software tool were tested with two levels of leaks and two different models with excess leaks (inspiration or expiration). The external software analyzed separately the inspiratory and expiratory unintentional leaks.

**Results:**

In basal condition, all ventilators but one underestimated tidal volume with values ranging between -1.5 ± 3.3% to -8.7% ± 3.27%. In the model with excess of inspiratory leaks, VT was overestimated by all four commercial software tools, with values ranging from 18.27 ± 7.05% to 35.92 ± 17.7%, whereas the ventilator independent-software gave a smaller difference (3.03 ± 2.6%). Leaks were underestimated by two applications with values of -11.47 ± 6.32 and -5.9 ± 0.52 L/min. With expiratory leaks, VT was overestimated by the software of one ventilator and the ventilator-independent software and significantly underestimated by the other three, with deviations ranging from +10.94 ± 7.1 to -48 ± 23.08%. The four commercial tools tested overestimated unintentional leaks, with values between 2.19 ± 0.85 to 3.08 ± 0.43 L/min.

**Conclusions:**

In a bench model, the presence of unintentional random leaks may be a source of error in the measurement of VT and leaks provided by the software of home ventilators. Analyzing leaks during inspiration and expiration separately may reduce this source of error.

## Background

Non-invasive home mechanical ventilation monitoring is an area of increasing interest. Built-in software has been designed for home ventilators, featuring on-line monitoring and downloading of data stored in the internal memory for later analysis. Although experts acknowledge that built-in software may provide important information [1], few data on its accuracy are available in the literature [2,3], and bench studies have shown a wide heterogeneity in the results obtained with the software designed by different manufacturers [4,5]. One of the main factors that may affect the reliability of monitoring in this setting is the presence of leaks in the system. Contal et al. reported that underestimations of the true volume by the ventilator are common in commercial software, with additional variability due to leak estimation [6]. Another study demonstrated a linear relationship between the presence of increasing leakage and volume underestimations [7].

To date, the relationship between leakage and monitoring in home ventilators has only been studied using linear models in which the leak is simulated by an orifice with a calibrated flow. This approach may not be representative of the leaks that occur in clinical practice [8], due to poorly fitting masks or to mouth exhalation in patients wearing nasal masks, because the magnitude of the leak may vary widely depending on the phase of the respiratory cycle (inspiration or expiration).

The present study aims to assess the accuracy of leak and tidal volume estimations provided by commercial home ventilators in a bench model designed to reproduce the clinical practice situation. We hypothesized that excess leaks only during inspiration or expiration (random dynamic leaks) may have an important effect on the estimations of unintentional leaks and tidal volume provided by the ventilator.

## Methods

### Study design

The experiment was conducted under simulation conditions in a mechanical ventilation laboratory equipped with a breathing simulator (Medical Ingmar SB 2000, Pittsburgh, PA, USA) which was used to test the home ventilators. As a signal acquisition system, an external polygraph (16Sp Powerlab, ADInstruments, Sydney, Australia), equipped with a pressure transducer (1050 model, ADInstruments), and a pneumotachograph (S300 model, instrumental dead space =70 mL, resistance = 0.0018 cmH2O/L/s, ADInstruments) was used. Sampling frequency was set to 200 Hz and the polygraph was connected to a personal computer equipped with Chart 7.0 software for Windows (ADInstruments).

**Figure 1:**
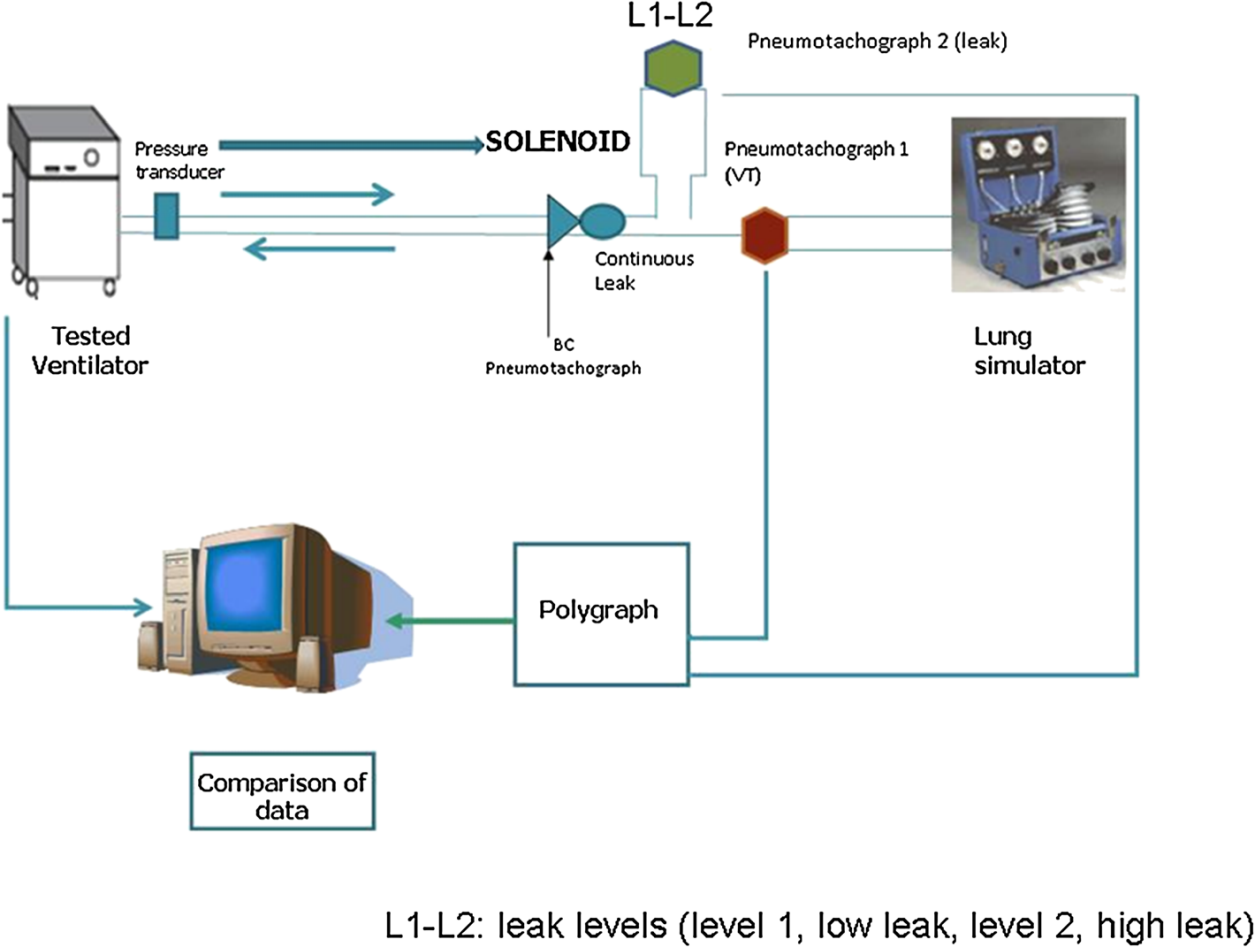
Design of the experiment (see text for more details).

The tested ventilators were connected to the simulator through single standard 2-m tubing, and a continuous leakage was placed at the distal end (Figure 1). A T-piece was inserted after the continuous leakage, and, in order to control the phase of the respiratory period in which unintentional leakages appeared, a solenoid valve (ADInstruments, Sydney, Australia) was attached to this T-piece. Opening and closing of the solenoid was electronically controlled through variations in the output voltage by the same software used to monitor leaks and tidal volume. The pressure wave generated by a transducer placed between the ventilator and the tubing acted as a trigger to open or close the solenoid (P1 in Figure 1). The tidal volume was monitored with a calibrated pneumotachograph at the simulator entrance (Pneumotachograph 1 in Figure 1).

Similarly, a second calibrated pneumotachograph was placed (Pneumotachograph 2 in Figure 1) distally to the solenoid valve in order to monitor leakage.

### Calibration

Before each session, both the pneumotachographs 1 and 2 (Figure 1) and the pressure transducer were calibrated following the manufacturer’s instructions, using a 3-liter syringe and against a water column respectively.

### Parameters used in the study

**Figure 2:**
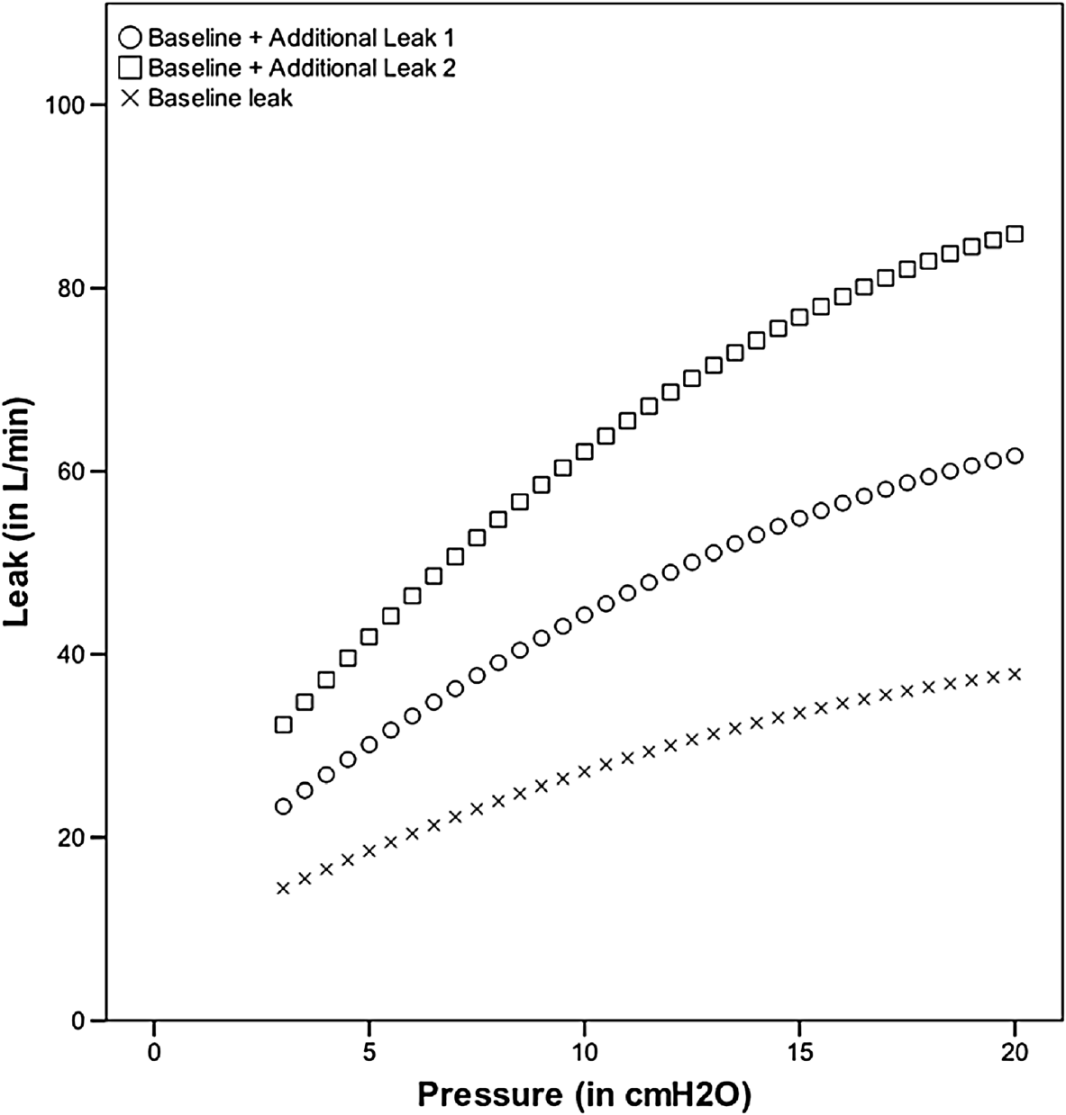
Pressure-leak plot of the basal and additional leaks.

a) Level of leak: In addition to the baseline intentional leak, corresponding to a standard nasal mask leak value, the ventilators were tested with two predetermined, calibrated leak flows (additional leaks 1 and 2 [low and high leak]) placed after the solenoid [8]. The level of baseline leak and additional leaks 1 and 2 are shown in Figure 2. The equations for the leaks used in the model were the following (R2 > 0.995 for all of them):

••Baseline (intentional) leak : Leak (in L/min) = - 0.0443p^2^ + 2.3943p + 7.659

••Baseline leak + Additional (unintentional) leak 1 : Leak (in L/min) = - 0.0737p^2^ + 3.9472p + 12.225

••Baseline leak + Additional (unintentional) leak 2 : Leak = - 0.1107p^2^ + 5.7015p + 16.179

b) Periods of leak: Two periods of leak were scheduled in the protocol: 1) a leak during the inspiratory phase, generated when the pressure reached 6 cm H2O, followed by the closure of the solenoid when expiration began; and 2) a leak during expiration, generated when the pressure fell to 6 cm H2O, and including the transition point towards inspiration, followed by the closure of the solenoid at the beginning of inspiration.

c) Parameters of the ventilator tested: Two different levels of pressure support (PS) were predefined:

••10 cmH_2_O (IPAP-15, and EPAP-5)

••15 cmH_2_O (IPAP-20 and EPAP-5)

Other parameters were fixed as follows: rise time was set at the intermediate value of 150 msec and cycle-off criterion at 30% of peak flow. The inspiratory trigger was set at the lowest sensitivity predetermined in each ventilator so as to avoid autotriggering. Minimum and maximum inspiratory time in the ventilators in which this parameter can be selected were switched off.

d) Parameters in the simulator: Spontaneous respiratory rate was programmed in the simulator at 15 breaths/min, with an effort level of 50% of the maximum allowed by the device, and corresponding to a pressure of -10 cm H_2_O and a duration of 50% of the inspiratory cycle. Compliance of 60 mL/cm H_2_O and resistance of 7.5 cm H_2_O/ L/sec were the mechanical patterns selected for the simulation.

### Software tested

Four commercial ventilators with their commercial built-in software from different manufacturers were tested: a) VIVO 50 (V50) (Firmware 2.0 General Electric, Mölnycke, Sweden); b) Puritan Bennett 560 (PB560) (Covidien, Mansfield. MA, USA); c) Trilogy 100 (T) (Philips Respironics, Murrysville, PA, USA); and d) Stellar 100 (St) (ResMed, North Ryde, Australia).

A fifth ventilator-independent software developed *ad hoc* for the present study was also tested (BetterCare, Sabadell, Spain – BC). The design of the *ad hoc* designed software is based on the following issues [9]:

Acquisition of waveforms: The used external software is based on the acquisition of pressure and flow signals from a disposable pneumotachograph (CO2/flow sensor, Philips Respironics) connected to a commercial monitor (NM3, Philips Respironics), which processed the data provided regardless of the ventilator used. In the model used in the present study, the pneumotachograph was placed after the 2-m tubing and immediately prior to the intentional leak (BC pneumotachograph in Figure 1).

Transference of waveforms: The acquired waveforms, pressure–time and flow-time were transferred to the software by means of the communication protocol of the NM3 monitor. Sampling of raw data was set at 200 Hz.

Estimation and correction of flow/time graphs for the intentional leaks: A test to calibrate intentional leaks through the leak valve was performed at the beginning of the evaluation of the external software, at rising values of CPAP from 4 to 20 cmsH2O, increased manually by occlusion of the distal end of the expiratory valve. A pressure-leak plot was constructed by the software approximating the system to a second degree equation. Leak testing was considered successful when the regression coefficient R^2^ was > 0.99. Intentional leaks through the valve were automatically subtracted from the flow-time waveform and did not compute in the leak waveform [7].

**Figure 3:**
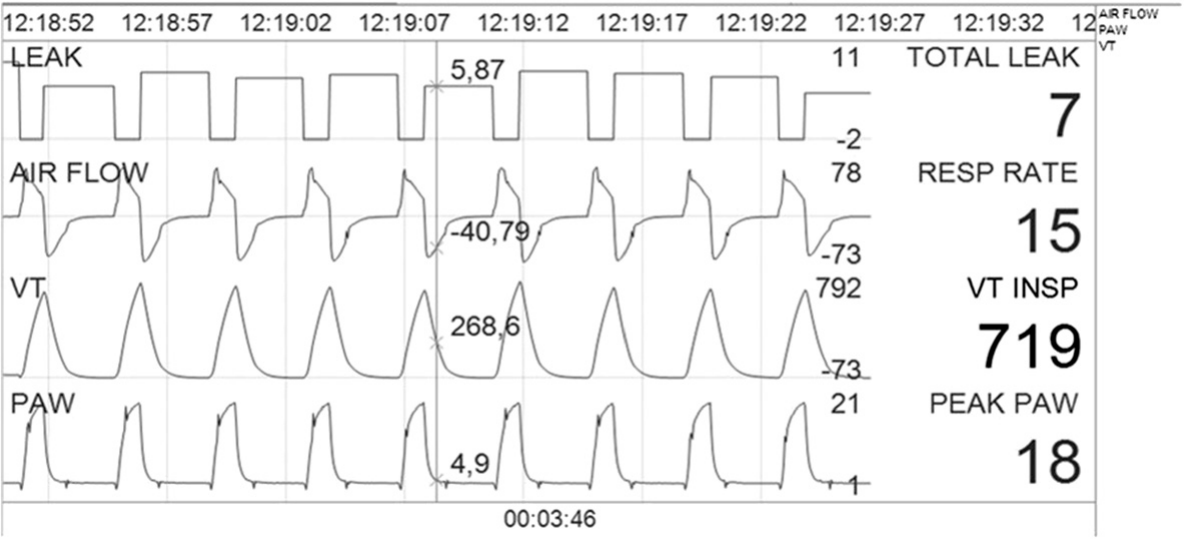
Display of the flow-time, pressure–time, volume-time graphs and unintentional leaks by the BC software in the model with expiratory leakage after correction of the flow-time graph for the intentional (by means of a leak test) and unintentional leaks (following the method of retrograde comparison of areas).

Estimation of non-intentional leaks: Non-intentional leaks (for this particular case, leaks escaping through the solenoid) were computed as follows: a) baseline expiratory leaks were captured from the flow waveform in the transition point from expiration towards inspiration. This transition point, and also the opposite point from inspiration to expiration, was determined by means of an automatic analysis of the pressure and flow graphs based on advanced iteration mathematical processing, that detected changes in the direction of the analyzed graphs. .In this transition point the flow of patients is considered to be zero, and the entire monitored flow through the pneumotachograph belongs theoretically to the leaks. The expiratory negative area under this point is considered as the expiratory tidal volume (ETV). After that point, the inspiratory phase is analyzed backwards, comparing the area under the inspiratory phase (Ai) and the area under expiration. If Ai > ETV, the difference between the two areas is considered to be the leak during the inspiratory phase. Conversely, if Ai < ETV, the difference was considered to be the leak during the expiratory phase. In both cases, the excess of volume, calculated as the difference between the areas under the flow-time waveform, was corrected by the inspiratory or expiratory time. The leaks were thus displayed as a biphasic waveform: mean leak during inspiration and during expiration (Figure 3). As the cycles were analyzed backwards, a time delay of 10 seconds was programmed between the acquisition of waveforms and their display on the screen, to prevent potential problems associated with cycles of different length.

### Measurement protocol

Two conditions were programmed for each step, with a scheduled duration of two minutes. In the first period the solenoid was kept closed, determining baseline differences in tidal volume and leak measurement found between the built-in software of the commercial ventilators and the external measurements. In the second 2-minute period, the solenoid was opened in the desired phase of the cycle, inspiratory or expiratory, generating a leak during inspiration or expiration.

Leaks and VT values of the last five breaths in each step were collected for analysis. Conversion to BTPS conditions: To standardize the analysis of true VT entering the respiratory system, the VT obtained by the pneumotachograph was converted to BTPS conditions [10] by applying the following formula:

$$\begin{array}{l} \mathrm{VT}\;\left(\mathrm{BTPS}\right)=\mathrm{VT}\;\left(\mathrm{ATPD}\right)\;\times \;(\mathrm{barometric}\;\mathrm{pressure}\\ \;-\mathrm{water}\;\mathrm{vapor}\;\mathrm{pressure}/\mathrm{barometric}\\ \;\mathrm{pressure}-47)\;\times \;310/\left(273\right)\\ \left(\;+\mathrm{temperature}\;\mathrm{in}\;{}^{\circ}C\right). \end{array}$$

Where 47 is the vapor pressure of H2O at 37°C, 273 is the temperature in Kelvin scale corresponding to 0° in centigrade scale; 310 is the value in degrees Kelvin at body temperature. Given that the room temperature at which the study was performed was 20° and the vapor pressure of H2O at that temperature is 17.5, substituting the values the conversion of ATPD to BTPS increased tidal volume by an average of 10.2%. As exclusion criteria, when the predefined leaks induced a continuous autotrigger the measures obtained were considered invalid.

### Statistical analysis

Statistical analyses were performed using SPSS version 19 for Windows (SPSS Inc., Chicago, IL, USA). VT and leak values for each experimental condition were expressed as means ± standard deviations (SD). Differences between estimated and measured leaks were expressed in absolute values in l/min, and differences between VT values provided by each software tested and the external system as absolute values in mL, and as a percentage of the externally measured VT, which was considered as the reference for the purposes of the study, with the formula:

$$\begin{array}{l} \left(\mathrm{VT}\;\mathrm{ventilator}–\mathrm{VT}\;\mathrm{pneumotachograph}\right)\\ \;*100/\mathrm{Vt}\;\mathrm{pneumotachograph}. \end{array}$$

The reliability of the measure provided by each software tested was evaluated using the intraclass coefficient correlation (ICC) for individual measures, for both baseline and leakage steps [11]. The externally measured VTs in the different predefined conditions, baseline and leak, were compared by means of non-paired Student’s t tests. The comparison between the software tested under the same controlled conditions was performed by means of ANOVA testing with Bonferroni post hoc analysis. The significance level was set at p <0.05 for all used tests.

## Results

Paired values of leak and VT were compared breath to breath. Overall, 300 cycles were included for analysis (150 cycles with baseline leak and 150 with additional leak). Differences in estimated VT at baseline intentional leak between different commercial ventilators are reflected in Table 1. As shown, mean deviation from the externally measured values never exceeded 10%, with an intraclass coefficient value of > 0.975 for all tested software.

**Table 1:** Difference in values displayed and measured (VT displayed-VT measured) in absolute values and percentages, and intraclass correlation coefficient (ICC) for individual measures for the five software tools studied (baseline conditions with intentional leak)

**Software**	**∆ Vt in mL**	**∆ Vt (%)**	**ICC**	**95% CI**	**p value**
Trilogy	-12 ± 24	-1.5 ± 3.3	0.985	0.965-0.992	<0.001
PB 560	-55 ± 24	-8.7 ± 3.27	0.978	0.953-0.989	<0.001
Stellar 100	-23 ± 23	-2.90 ± 2.8	0.980	0.958-0.990	<0.001
VIVO 50	-31 ± 16	-4.5 ± 2.05	0.993	0.984-0.996	<0.001
Bettercare	24 ± 16	3.46 ± 2.22	0.992	0.982-0.996	<0.001

Δ mL = Vt ventilator – Vt measured.

% Vt = (Vt ventilator – Vt measured) *100 / Vt measured.

Regarding leak estimation, values of the cycles with the solenoid valve closed and baseline leak were, for software displaying total leak, 27.48 ± 2.42 L/ min (T), and 27.81 ± 1.59 L (V50). For software displaying only unintentional leakage, baseline values were 2.03 ± 0.8 L/min (PB 560) and 0 L/min (St). A measure of 0 L/min was also provided by the ventilator-independent software tested (BC).

### Model with an inspiratory additional leak

#### Accuracy of VT estimation

**Figure 4:**
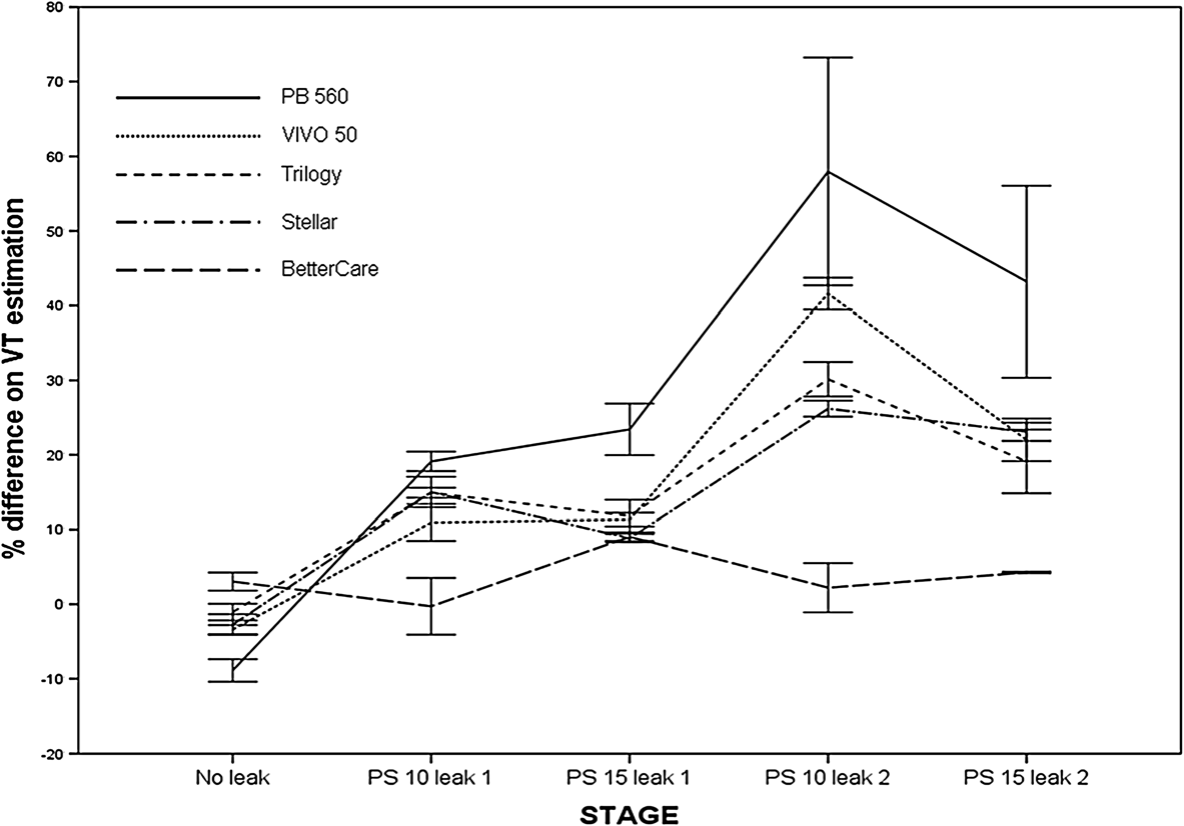
**Effect of inspiratory additional leakage on the accuracy of VT estimation in the different steps of the protocol.** Significant differences in percentage of VT were found at different stages for all tested software, with the exception of ventilator-independent BC software (p < 0.001 ANOVA test). Differences were also found between the different software tools tested (p < 0.001 ANOVA test). See text for more details.

When comparing the VT values provided by the built-in software of the commercial ventilators with the externally measured values in cycles with inspiratory additional leaks, it was found that the commercial software tested significantly overestimated VT. As shown in Figure 4, significant differences were detected when analyzing the five combinations of pressure support values and levels of controlled leak; when the software were individually analyzed, the overestimation was greater when higher leak levels were predetermined for all but one (BC) software (p < 0.01 ANOVA test). Overall mean absolute differences (including all cycles with excess of inspiratory leak) ranged from 137 ml (18.27 ± 7.05%, St) to 264 ml (35.92 ± 17.7%, PB 560). The ventilator-independent software (BC) presented a difference of 23 ± 20 ml from the external measure, corresponding to 3.03 ± 2.6% of baseline VT, and a difference of 29 ± 28 ml, corresponding to 3.81 ± 3.9% of the externally measured VT with inspiratory leakage. Overall, no differences regarding overestimation were detected between T, St and V50, whereas mean differences were detected between BC and the other four ventilators. (p < 0.01 ANOVA test for the comparison between different software). Table 2 reflects the reliability analysis of the Vt estimation in the cycles with inspiratory leaks for all software tested.

**Table 2:** Reliability analysis (intraclass correlation coefficient for individual measures) for the Vt estimation in cycles with excess of leaks during inspiratory and expiratory cycle

	**Excess of inspiratory leak**			**Excess of expiratory leak**		
**Software**	**ICC**	**95% CI**	**p value**	**ICC**	**95% CI**	**p value**
Trilogy	0.938	0.852-0.975	<0.001	0.840	0.481-0.958	<0.001
PB 560	0.687	0.362-0.863	<0.001	0.976	0.908-0.994	<0.001
Stellar	0.932	0.837-0.973	<0.001	0.969	0.882.-0.992	<0.001
Vivo 50	0.652	0.306-0.846	<0.01	0.713	0.492-0.929	<0.01
BetterCare	0.972	0.930-0.989	<0.001	0.997	0.986-999	<0.001

**Table 3:** Mean unintentional leaks (in L/min) registered after the solenoid valve at each stage of the study

	**Inspiratory phase leak**	**Cycle leak**	**Expiratory phase leak**	**Cycle leak**
PS 10 leak 1	13.84 ± 0.87	6.94 ± 0.62	9.8 ± 1.38	6.21 ± 1.02
PS 15 leak 1	16.33 ± 1.01	8.23 ± 0.61	9.6 ± 1.16	6.05 ± 0.9
PS 10 leak 2	25.07 ± 3.3	12.42 ± 2.04	----------------	--------------
PS 15 leak 2	29 ± 5.23	14.74 ± 2.84	----------------	---------------

Cycle leak = Phase leak /Total cycle time.

#### Accuracy of leak estimation

**Figure 5:**
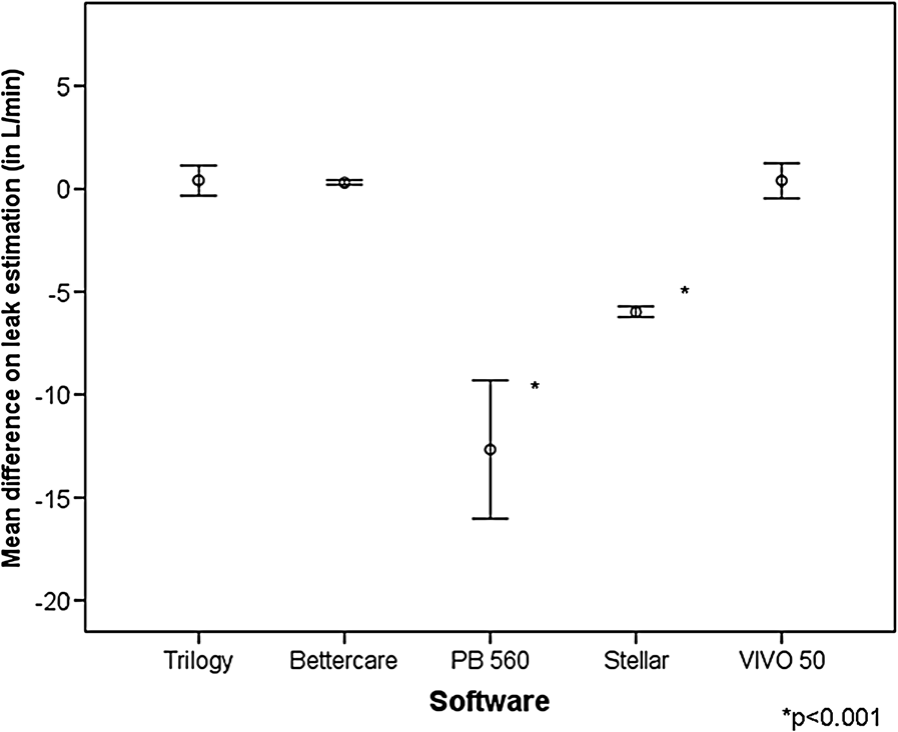
**Accuracy of the measurement of the excess of the inspiratory leakage for each software tested, with points displaying the mean deviation in L/min and bars the 95% CI.** Significant differences (p < 0.001) were detected for St and PB 560.

**Figure 6:**
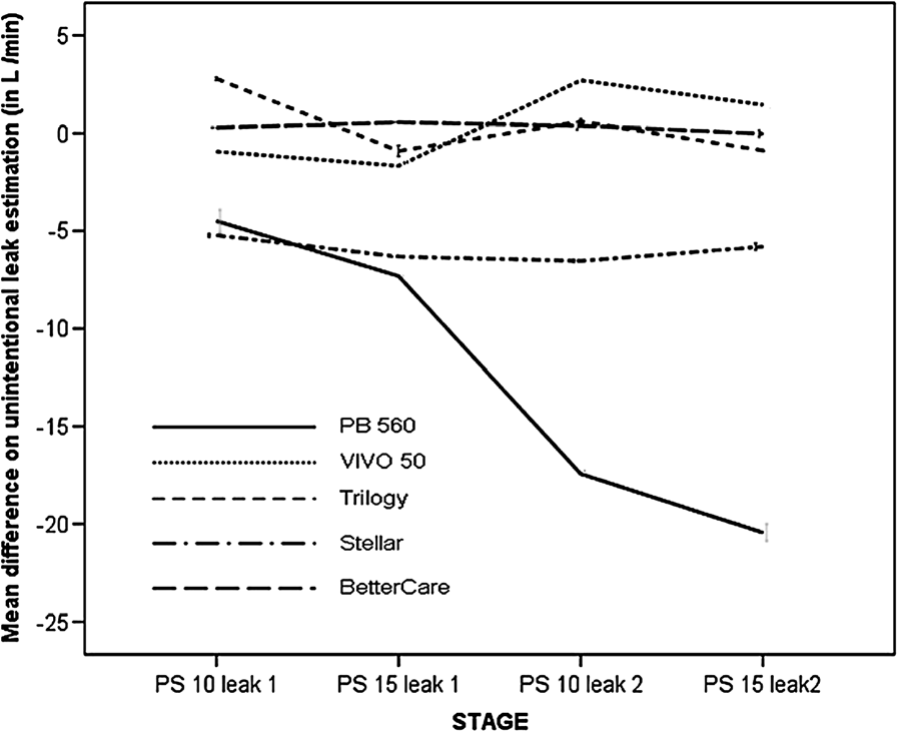
Effect of inspiratory additional leakage on the accuracy of unintentional leaks estimation in the different steps of the protocol.

The mean amount of unintentional phase and entire cycle leaks through the solenoid valve are reflected in Table 3. When compared with the estimated values provided by the commercial software, as shown in Figure 5, two of them (V50 and T) and the ventilator-independent software (BC) presented a cycle to cycle mean difference below 1 L/ min when compared with the external measurement for their estimation of leaks (p = ns). The other two commercial software (PB 560 and St) significantly underestimated the leak, with overall values of -11.47 ± 6.32 l and -5.9 ± 0.52 L/min. Figure 6 displays the mean difference of estimation in each step of the protocol. As shown, a progressive underestimation of the unintentional leaks with increasing values is found in one of the software. With the commercial software tools (V 50 and T) that display leakage on the screen as a biphasic wave with inspiratory and expiratory leaks both externally measured inspiratory and expiratory values were higher, although the true unintentional leakage occurred only in the inspiratory part of the cycle, as identified by the external measurement used in the study. Mean increases in expiratory leakage were 2.4 ± 3.9 for T and 2.8 ± 4 l/min for V 50. The ventilator-independent software (BC) did not detect any additional expiratory leak, as it only identified the inspiratory leakage.

### Model with expiratory leaks

The addition of a high expiratory leak corresponding to level 2 in the bench experimental model induced autotriggering in all the commercial ventilators tested. For this reason, only cycles with a leak at level 1 were included in the analysis.

#### Accuracy of VT estimation

The addition of expiratory leaks decreased the measured tidal volume from 665 ± 125 ml to 585 ± 103 ml (p < 0.001). No differences were found between the commercial ventilators tested.

**Figure 7:**
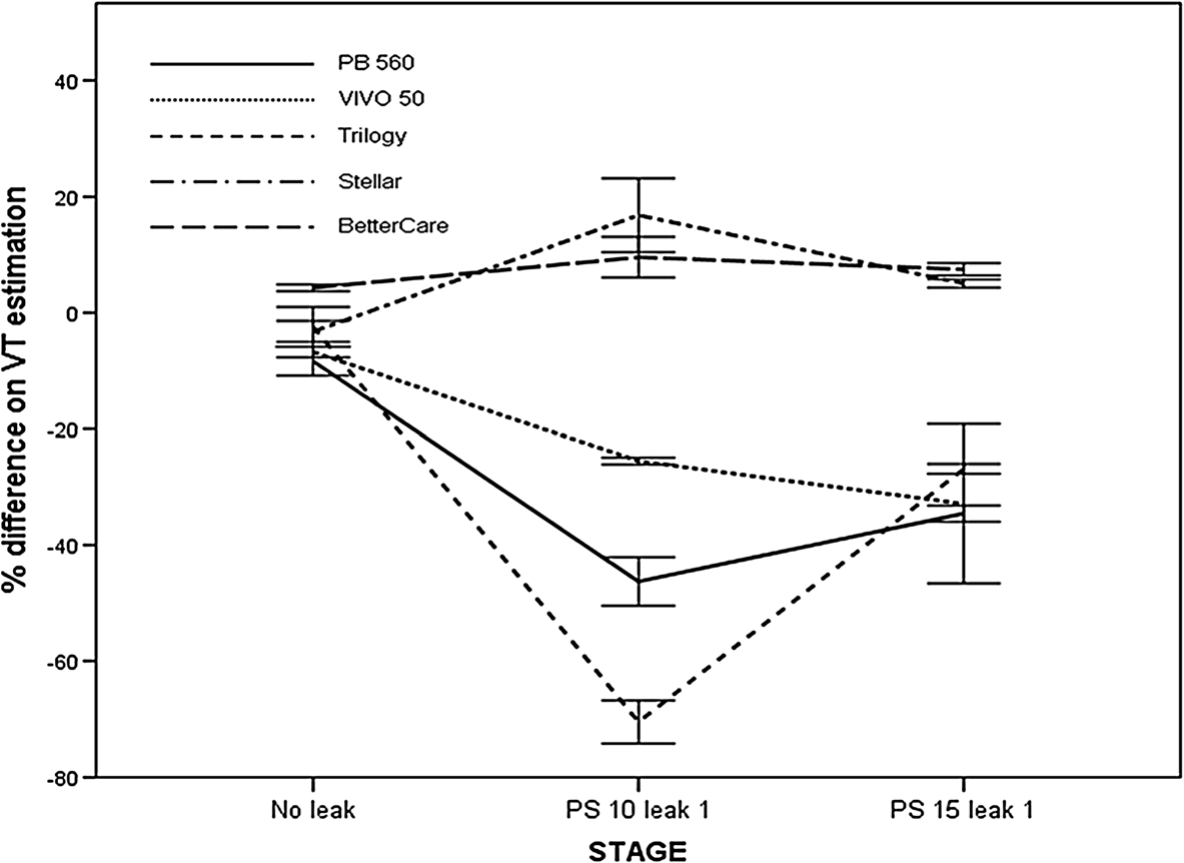
**Effect of expiratory additional leakage on the accuracy of VT estimation.** The addition of expiratory leak resulted in underestimation of VT by three software, and overestimation in the remaining two. Significant differences were found between tested software with an excess of expiratory leak (p < 0.001 ANOVA test).

When comparing VT values provided by built-in software with externally measured values in cycles with expiratory additional leaks, three software tools (T, PB 560 and V50) significantly underestimated VT, with values ranging from -48 ± 6.7% (T) to -29 ± 7.5% (V 50). The remaining built-in software (St) overestimated VT by 10.94 ± 7.1%, and a similar overestimation (8.53 ± 2.26%) was found with the ventilator-independent software (BC). Figure 7 represents mean values with and without expiratory leakage. Significant differences were found between stages for each individual software tool (p < 0.05, ANOVA). The addition of expiratory leakage decreased the reliability of the measures, as reflected in Table 2.

#### Accuracy of leak estimation

**Figure 8:**
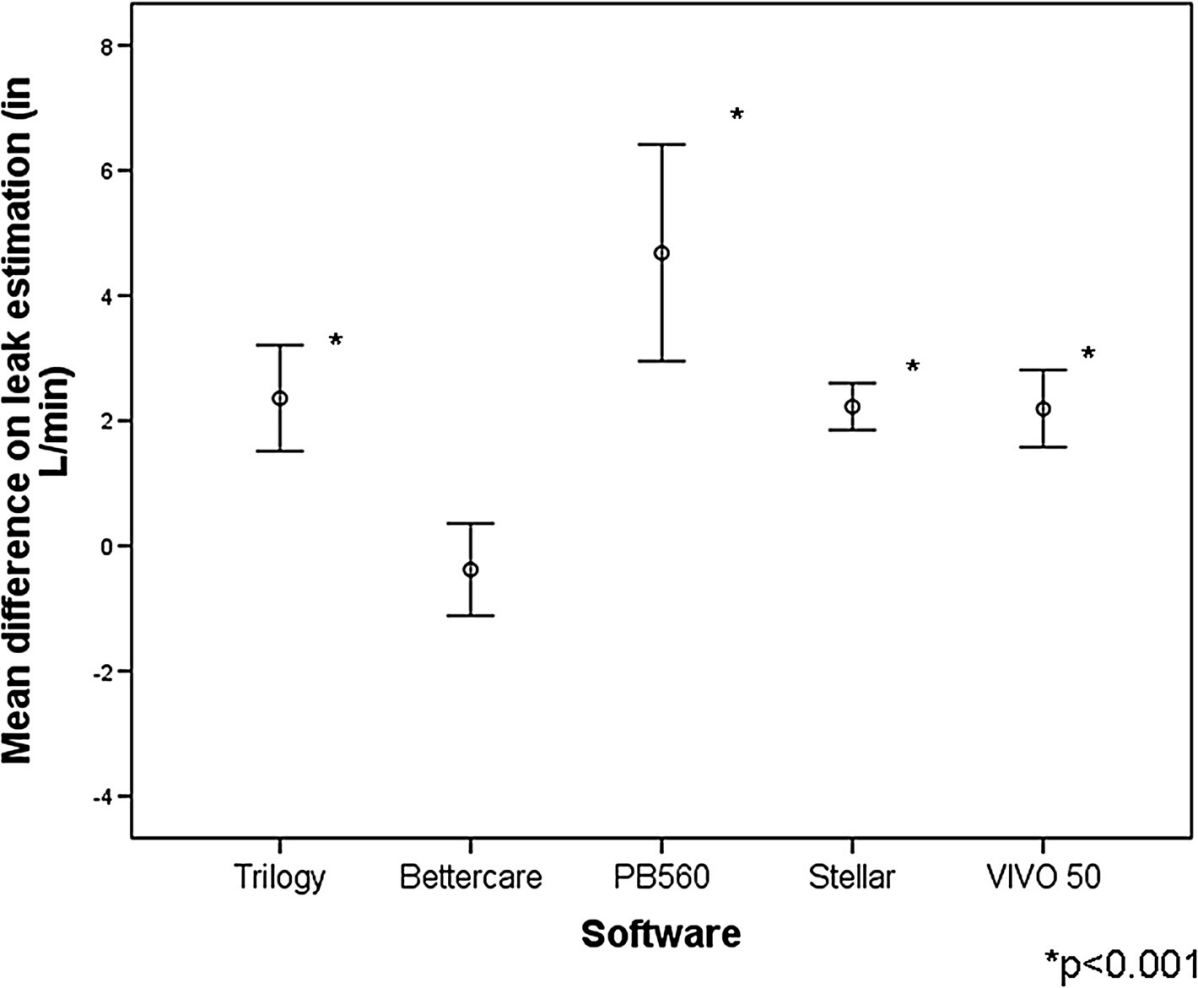
**Accuracy of the measurement of the excess of expiratory leakage for each tested software, with points displaying the mean deviation in L/min and bars the 95% CI.** Significant differences (p < 0.001) were detected for all software except for the ventilator-independent BC software.

**Figure 9:**
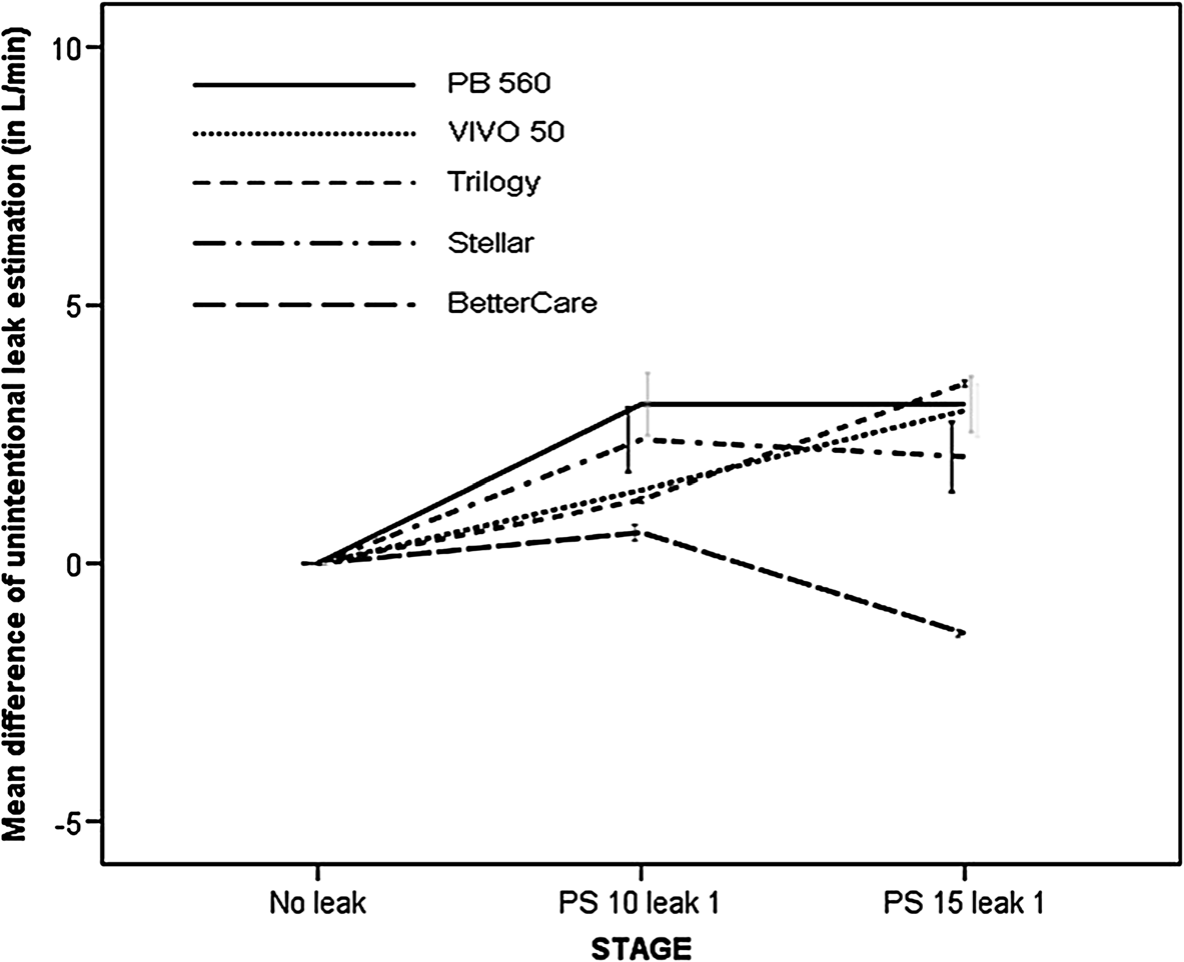
Effect of expiratory additional leakage on the accuracy of unintentional leaks estimation in the different steps of the protocol.

As shown in Figures 8 and 9, all software tools significantly overestimated the leaks, with values ranging from 2.19 ± 0.85 L/min (V50) to 3.08 ± 0.43 L/min (PB 560). As in the case of the inspiratory model, software displaying leaks as a biphasic wave on the screen increased the values of inspiratory and expiratory leakage. The ventilator-independent external software slightly underestimated values of leakage with a difference of -0.38 ± 1.03 L/min with respect to the reference value used (p = ns).

## Discussion

This study shows that, in a bench model, the presence of random dynamic leakage may be a source of error in the estimation of VT and leaks, which are two of the most important variables required for monitoring non-invasive ventilation. This error emerged to be more important for Vt estimation. Moreover, when results obtained with the built-in software of four commercial ventilators were compared with a ventilator-independent external software designed *ad hoc* for this purpose, the ventilator-independent external software estimated both VT and leaks more accurately, suggesting that the bias identified in the four commercial ventilators was attributable to the built-in software.

A similar model of leakage, which is greater during inspiration than during expiration, were used by Carteaux et al., using a water column of variable height to study the frequency of asynchronies at different leak levels [12]. The impact of these models on monitoring reliability, however, has not been evaluated to date. Interestingly, in their study Carteaux et al. found an increased incidence of autotriggered breaths with continuous leakage. In our model, although not designed for this purpose, we also found autotriggering in the presence of leaks, with a clearly higher incidence in the model with expiratory leak. This phenomenon prevented us from completing the tests with the highest expiratory leak.

Studies of the reliability of parameters provided by built-in software of commercial ventilators have used a calibrated orifice to simulate the leak [13,14], and have demonstrated a systematic underestimation of VT with this design. For example, Contal et al. demonstrated highly variable levels of underestimation with different ventilators, which ranged between -89 and -328 ml and were independent of the magnitude of the leakage [6]. These authors also found significant differences in the estimated leaks between different built-in software. Following a very similar model, our group demonstrated that in this setting the relationship between incremental values of leakage and the underestimation of volume shows a linear pattern. This phenomenon can be explained by the linear loss of pressure along the tubing, and may be corrected by incorporating algorithms that calculate this loss of pressure as a function of the exiting flow [7].

The reasons for the potential source of error in the estimation of VT and leakage under the assumption of non-linear random dynamic leakage should be analyzed in detail. In a single-tube system with intentional leakage, the pneumotachograph inside the ventilator monitors total gas flow, which corresponds to VT and unintentional and intentional leaks. In each cycle it must discern the amount of gas corresponding to VT and the amount corresponding to unintentional leaks, assuming that the intentional leak in the circuit is known [15]. In our study, in the model with inspiratory leak, which we consider more reliable from the clinical point of view, the four ventilators with built-in software, contrarily to the data provided by previous articles (6,7), significantly overestimated VT, suggesting that a portion of the identified leak was erroneously considered by the software as volume delivered to the patient. This hypothesis is supported by the finding that, although with variations between ventilators, the error increases with higher leaks and level of pressure support (Figure 4). This finding also accounts for the underestimation of the leakage observed in two of the commercial ventilators assessed. The built-in software of the other two commercial ventilators, however, showed only a minimal deviation from the externally monitored values in the leakage. This may be because these software tools detected increases in both the inspiratory and expiratory phases of the cycle, when the leak was simulated only in the inspiration. In fact, if we consider only the variation in inspiratory leakage, the software of these two ventilators also underestimated the leak. In the same model, using ventilator-independent external software that separates inspiratory and expiratory leakages, smaller errors in the estimations of both VT and leakage were found.

We found an underestimation of VT in the model with expiratory leaks in three of the commercial ventilators studied. This suggests that the software gave a higher estimation for the leakage than the externally measured reference and for this reason underestimated the VT. However, this hypothesis does not fully account for the findings during the assessment of the Stellar ventilator; although its leak overestimation was in a range similar to the other ventilators tested, it also showed a slight tendency to overestimate VT in the measures provided. The use of ventilatory-independent software separating inspiratory and expiratory leakages in this case, too, reduced the error in the estimated VT and leaks.

The findings of our study may have clinical implications, because an excess of leaks exclusively during the inspiratory phase is a common situation in clinical practice. In this setting, all commercial software overestimated VT and some underestimated the real leak. These inaccuracies may lead the clinician to misinterpret the underlying causes of patient-ventilator interactions.

The model used for the design of the ventilator-independent external measuring system, despite its higher reliability with this model of leakage as shown by the bench test, may also have some limitations. The first is that it assumes the leakage flow to be constant during the expiratory phase, a situation that may not happen in all patients. Accordingly, this model must be validated in clinical practice, especially in situations with patient-ventilator asynchrony. For example, double-triggered cycles may overestimate leakages transiently in the first cycle, because expiration is incomplete. The model may also need adjustments in breaths presenting air-trapping, in which inspiratory and expiratory areas may differ. Finally, this is an experimental model, and the picture in clinical practice may be much more complex. For example, patients with active expiration, fighting against the ventilator or with an abrupt upper airways occlusion may present different patterns of leaks.

## Conclusions

Random dynamic leaks can be a source of error in the estimation of the VT and unintentional leaks provided by the built-in software in commercial home ventilators. Analyzing leaks during inspiration and expiration separately may reduce this source of error.

## Abbreviations

Ai: Area under the inspiratory phase; BC: Better Care; CI: Confidence interval; EPAP: Expiratory pressure; ETV: Expiratory tidal volume; ICC: Intraclass coefficient correlation; IPAP: Inspiratory pressure; PB560: Puritan Bennett 560; PS: Pressure support; SD: Standard deviation; St: Stellar 100; T: Trilogy 100; V50: VIVO 50; VT: Tidal volume.

## Competing interests

Conflict of interest: AS, JM, XP, ML and EM: none declared. LB: has developed patented inventions related to monitoring ventilator signals. The license for these patents belongs to Corporació Sanitària Parc Taulí – Institut Universitari Parc Taulí, Spain. LB own 10% of BetterCare S.L., a research and development spin-off of Corporació Sanitària Parc Taulí, Spain.

## Authors’ contributions

AS and ML designed the study, carried out the measurements and calibrations, interpretation of data, statistical analysis and wrote the first draft. JM designed the ad hoc software. LB designed the ad hoc software and corrected the first draft. XP carried out the measurements and calibrations. EM contributed to the designed the study and statistical analysis. All authors read and approved the final manuscript.

## Pre-publication history

The pre-publication history for this paper can be accessed here:

http://www.biomedcentral.com/1471-2466/13/75/prepub
